# Why are voluntary health insurance holders willing to pay tax towards public healthcare? A qualitative study from Sweden

**DOI:** 10.1186/s12913-026-15708-3

**Published:** 2026-09-29

**Authors:** Linn Kullberg, Paula Blomqvist, Ulrika Winblad

**Affiliations:** 1 Department of Public Health and Caring Sciences, Uppsala University, Box 564, Uppsala, SE-751 22 Sweden; 2 Department of Government, Uppsala University, Box 514, Uppsala, SE-751 20 Sweden

**Keywords:** Voluntary health insurance, Tax-funded health system, Willingness to pay tax, Sweden, Thematic analysis

## Abstract

**Background:**

Voluntary health insurance (VHI) is increasing in many European healthcare systems, including the tax-funded public healthcare systems found in Northern Europe. As private alternatives to publicly financed health services expand, there is a risk that the willingness to pay tax towards redistributive public healthcare systems will diminish. This study investigated how employer-funded VHI holders accounted for their continued willingness to pay income tax to suppor the public healthcare system and the motivation underlying their tax compliance.

**Methods:**

A qualitative approach based on 19 semi-structured interviews with individuals holding an employer-funded VHI in Sweden was used. The interviews were analysed using thematic analysis.

**Results:**

The findings suggest a continued willingness to pay tax towards the public healthcare system among Swedes with an employer-funded VHI. The support was motivated by a moral logic and belief in the principle of equal access to health for all citizens. Respondents also expressed ambivalence about gaining faster access to care than others.

**Conclusion:**

These accounts suggest that the growth of employer-funded VHI does not by itself erode the willingness to fund public healthcare, and that the egalitarian principles underpinning a universal system can sustain a sense of obligation to contribute even among those who use private healthcare. The ambivalence respondents expressed indicates that contributing on egalitarian grounds and benefiting from privileged access are not easily reconciled.

**Supplementary Information:**

The online version contains supplementary material available at https://doi.org/10.1186/s12913-026-15708-3.

## Background

Public healthcare systems are built on the idea of redistribution and financial protection. Health services are available to everyone, not only those who can afford to pay at the point of use. This redistribution occurs both between individuals and over time, ensuring a more equitable distribution of costs. Typically, financing is structured through taxation or social insurance [1]. The redistributive effect is particularly pronounced in tax-based, universalistic healthcare systems, where all citizens contribute to a single risk pool operated by the state, ensuring equal access to care regardless of individual financial contribution, employment status, or other distinguishing criteria [2]. In tax-funded universal healthcare systems, *medical need* is the only principle behind the distribution of health services. However, this principle of distribution is challenged by the rise of voluntary health insurance (VHI) in tax-based healthcare systems, as seen in the UK and the Nordic countries [3, 4], where the distributive logic is based on access to a privately purchased insurance rather than medical need.

VHI is distinguished from public healthcare systems in the European context in several ways. Firstly, it is voluntary in nature [5, 6]. Secondly, it is primarily funded privately, either by individuals or employers. Thirdly, it relies on risk-based premiums rather than income-based contributions [5, 7]. Except in the Netherlands, Switzerland, and Ireland, VHI plays a minor role alongside public systems that cover the majority of the population [8, 9]. VHI is typically categorised into different types based on its role alongside the public healthcare system. In tax-funded systems offering comprehensive services, *duplicating* VHI is the most common type, covering services already included in the public system but providing faster access and greater choice of providers. However, *complementary* VHI also exists in these systems to a lesser extent [9, 10].

Apart from undercutting the principle of distribution based on medical needs, the increase in VHI has also raised concerns that those who have it might become less willing to contribute to public healthcare systems, as they are paying for and consuming at least some healthcare services through the private market [11]. The public’s support for this solidaristic idea and their willingness to pay taxes or other social fees to sustain it are crucial for the functioning of public healthcare systems [12].

As tax-based healthcare systems typically make it impossible to “opt out” of them, this means that individuals with VHI are “paying twice” – at least for some services and to some extent. Some have also argued that gaining access to a private market of care providers, through a VHI, could result in an unfavourable comparison with public healthcare services, thus leading to a more critical view of the public system and reduced willingness to pay a tax towards it.

So far, research on the effects of holding VHI on support for and willingness to pay tax towards public healthcare in Europe is limited and inconclusive. While some studies suggest that having VHI is associated with reduced support for public healthcare systems and willingness to finance them [13], there are also studies from the Nordic countries that found no evidence of diminished support for [14], or reduced willingness to pay tax towards public healthcare systems [15]. Although the empirical literature is still scarce, these results raise the question of why VHI holders, at least in some cases, appear willing to continue to pay tax towards public healthcare systems and what their motives for this are.

Sweden represents a case of a tax-funded healthcare system where VHI has grown markedly over the past decades [15]. Currently, about 15 per cent of the employed population holds VHI [16], with most policies being employer-funded and concentrated in the private sector, where coverage rates reach up to approximately 40 per cent in certain industries [17]. Despite its growth, VHI accounts for only about 1 per cent of total health expenditures [18].

Given Sweden’s legacy of an egalitarian and tax-funded healthcare system, the growth of VHI among the middle class raises concerns about its impact on solidarity and equity [15, 19]. Employer-funded VHI holders, predominantly from the middle and upper-middle classes, are particularly relevant to study, as their support is crucial for the legitimacy of the public system [20]. These groups not only contribute significantly to public funding but also possess the financial means to supplement public services with private solutions like VHI [21]. Notably, the majority of VHI holders in Sweden receive their insurance as an employment benefit, often without actively choosing it due to dissatisfaction with public services. This means that the additional cost for a VHI is relatively small, as the employer covers the majority of the premium.

The relevance of this study lies in understanding whether holding VHI affects individuals’ willingness to fund the public system, which is key to assessing the risks of a dual healthcare model that could undermine public solidarity and erode the foundation of Sweden’s equity-oriented healthcare system. The central question is whether access to a private healthcare market outside the public system makes employer-funded VHI holders less willing to pay tax towards this system, or, conversely, what motivates them to continue supporting it despite holding VHI.

In this article, these questions were investigated through an empirical study based on interviews with 19 individuals holding an employer-funded VHI, who were asked about their view of the public healthcare system and their willingness to pay tax towards it. The respondents described a continued willingness to pay tax towards public healthcare and did not present the insurance as having reduced it. Their willingness was framed primarily in moral terms, with equal access to healthcare for all citizens invoked as the central value. Respondents also expressed ambivalence about the preferential treatment their own insurance affords, which sat uneasily with the principle they gave as their reason for contributing.

The rest of the paper is organised as follows. First, previous research on the effect of VHI on support for public healthcare is reviewed, followed by a theoretical section describing the main motives for paying tax: self-interest and values. Thereafter, the Swedish case study setting and methodological approach are presented, followed by the empirical results. The findings are summarised and discussed in the concluding section.

### The willingness to pay tax towards public healthcare

The legitimacy of European healthcare systems rests on a normative belief in public responsibility for the provision of health services to the population and a willingness to pay tax (or other social fees) to this end [12]. European attitudinal surveys have documented that such normative values are widespread in most European countries [22].

Earlier studies suggested that citizens in tax-funded, NHS-type, systems were generally more supportive of public involvement in healthcare [23, 24]. More recent research, however, has analysed institutional factors separately rather than grouping them by healthcare system type. These studies indicate that citizens in healthcare systems with greater state involvement [25] and broader public coverage of services [26] tend to show higher levels of support for public healthcare. A greater share of publicly funded healthcare has also been linked to stronger support for government involvement in the sector [27–29]. Similarly, higher private out-of-pocket expenditures are associated with lower evaluations of the healthcare system [27].

A recent study examined the effects of privatisation reforms on public attitudes towards healthcare systems in Europe. The findings highlighted a temporal dimension: initially, such reforms led to more positive evaluations, as they were perceived to enhance choice, but over time, this effect diminished. Instead, privatisation became associated with increasingly negative perceptions of the healthcare system, particularly as it led to reduced access and higher personal costs. The authors suggest that personal experiences of healthcare access may be a key explanatory mechanism for this shift [30].

In addition to attitudes towards the role of government in healthcare, previous research has also investigated the willingness to pay tax to fund public healthcare. A commonly used indicator is a question from the *International Social Survey Programme* (ISSP, round 2011, Health) asking, “*How willing would you be to pay higher taxes to improve the level of healthcare for all people in [your country]?*” (see, for instance, [31–33]. Several factors have been found to be associated with such willingness. First, *socioeconomic* factors such as income and education seem to matter, and having a university degree increases the willingness to pay a tax to improve healthcare [34]. Another factor is age. Barnes et al. [35] found that young individuals in the UK preferred lower levels of taxation compared to older persons. When it comes to income, the evidence is inconclusive. In some studies, higher income was found to be associated with greater willingness to pay tax towards public healthcare [32, 34], while others have found the opposite pattern [36]. Lower health status has also been found to correlate with greater willingness to pay a healthcare tax [34].

Another factor that has been found to affect the willingness to pay healthcare tax is political orientation or partisanship. Individuals oriented towards the political left [34] or those who agree with egalitarian values [32] have been found to be more likely to support increased taxation towards public healthcare. Similarly, Maldonado et al. [31] found that individuals describing themselves as having “humanitarian” values had a higher willingness to pay tax towards public healthcare, particularly in the case of high-income earners [31].

#### VHI and willingness to pay tax towards public healthcare

During the last decades, the role of VHI has grown in many countries, which has also led to a larger sector of healthcare services provided exclusively to patients with such insurance [37, 38]. In the wake of these developments, concerns have been raised regarding the preservation of solidarity and willingness to pay tax towards public healthcare in Europe. As a result, a growing body of literature is focusing on the impact of privatisation on public opinion and support for public healthcare [39–42]. As already noted, individuals in countries with a larger share of privately funded healthcare tend to be less supportive of governmental spending on healthcare [22, 23, 25, 29]. Zhu and Lipsmeyer suggest that this predominantly reflects the attitudes of upper- and middle-class groups in such systems, as they, to a higher degree, bear a financial burden for the healthcare needs of other people while at the same time often paying for their healthcare privately [29]. This suggests that an increased use of VHI will reduce the willingness to pay tax towards the public healthcare system.

In tax-based systems, VHI implies a sort of “double payment” for some healthcare services, as insurance holders continue to pay income tax towards public healthcare services while at the same time paying privately for VHI. This may be true, albeit to a lesser extent, even if the VHI is obtained as an employment benefit, as it may still contain deductibles and a fringe benefits tax. The benefits of having a duplicating VHI in a universalistic, tax-based system may include faster access to care and access to services which are perceived to be of higher quality than those in the public system [43]. Both the double payment and the exposure to the private market for healthcare services may lead to a reduced willingness to pay tax towards the public system [11].

There are relatively few studies that have empirically examined the effects of having VHI on the willingness to pay tax towards public healthcare in tax-based healthcare systems. A British study, based on survey data, found no difference between those having VHI and the general population in support for existing levels of government spending on healthcare but showed that those who were privately insured were less positive towards *increased* spending [44]. However, another British study found VHI holders to be less supportive of public spending on healthcare than non-holders [45].

In the Scandinavian context, Martinussen and Magnussen [14] investigated the association between having VHI and attitudes towards public healthcare using data from a Norwegian national survey. They did not find any differences between VHI holders and non-holders regarding attitudes towards governmental responsibility for healthcare, but noted that those with VHI were more positive towards commercial actors in healthcare. In Sweden, Kullberg et al. [15] investigated the association between having VHI and willingness to pay tax towards public healthcare. After controlling for income, education, and political orientation, they found no differences between those having VHI and others regarding willingness to pay tax towards public healthcare, suggesting that VHI may act as an intermediate factor rather than having an independent impact on willingness to pay tax In sum, there have been some concerns that a rise in VHI might reduce willingness to pay tax to support public healthcare, as citizens in countries with a high share of privately financed healthcare have been shown to be more negative towards government spending in this area. The empirical findings on this matter are, however, inconclusive, particularly in the Scandinavian context, where no such pattern has been found. In the next section, different motives for paying a healthcare tax are reviewed.

### Motives for paying tax

Why people consent or are willing to pay tax is the central question in the literature on *tax compliance* [46, 47]. In the case of paying tax towards social welfare, including healthcare services, two main categories of motives have been identified: motives based on self-interest and motives based on norms and values [48–51].

Motives based on self-interest are grounded in rational choice theory and the idea of *homo economicus* – namely, that a human being is inherently a rational actor, acting out of economic interests [50]. From this perspective, preferences regarding taxation are guided by how the particular policies funded by the taxes are expected to benefit the individual in question. In line with this, it can be expected that high-income earners are less supportive of public welfare programmes and progressive taxation than others as they are likely to be net contributors [12, 32, 36, 50, 52, 53]. In the case of healthcare, self-interested individuals can also be expected to consider their own estimated need for healthcare services. If this need is expected to be low, either because they see themselves as healthy or because they have access to privately financed healthcare services, willingness to pay tax towards public healthcare is likely to be reduced. On the other hand, if they have high health needs and not all healthcare services are covered by their VHI, it can still be rational for a VHI holder with a relatively high income to support publicly funded healthcare, [12]. The concept of self-interest has been widened to also apply on the *group level.* In such cases, individuals might display a willingness to pay tax for public services if they expect that these will benefit members of their own group, such as family members or friends, even if they themselves will be net contributors or expect no gain from them [53].

The other category of motives for tax compliance draws on the idea of a *homo sociologicus*, according to which human actions are motivated by norms and values [50]. According to Steele and Breznau [53], values refer to “principles of behaviour or normatively desirable outcomes” (p. 4), such as egalitarianism or justice. They can lead individuals to act in opposition to their material self-interest – for instance, by supporting higher taxes – if this is supported by their moral convictions [53]. One moral value which has played a central role in the construction of European healthcare systems is *solidarity* [54, 55]. Ferdman and Kohn [56] define solidarity as a concept that links the social, political, and moral together, where feelings of community can motivate individuals to place the needs of others above their own interests, or to see interests as something collective. The borders of the community might vary, as the central idea is the feeling of unity that ties people together and makes them identify with the collective rather than pursue their self-interests. Lynch and Kalaitzake point to the origins of solidarity as impulses to nurture and support vulnerable others, describing them as “secondary forms of caring”. Solidarity, they argue, is “a macro-level expression of collective caring, or politicised form of love” [57]. In this paper, the difference between group-based self-interest and solidarity is that group-based self-interest refers to caring about people one knows *personally*, such as family members or friends, while solidarity refers to a larger collective with whom one has no personal affiliation, such as a wider community.

Related to solidarity, but distinct from it, are motives related to the value of *egalitarianism.* Egalitarianism, i.e., the idea that all individuals should be treated in an equal manner, can be related to different things, such as all humans having equal value; the idea that all people should enjoy the same political, economic, social, and civic rights; or a position that favours removal of economic inequality through redistribution of income [32]. Applied to healthcare, egalitarian values imply that all individuals should have equal access to healthcare, regardless of their economic, social, or health-related status. This implies that healthcare services should not be distributed through markets but by governments honouring such principles [58]. According to Feldman and Steenbergen, egalitarianism refers to a belief in the value of equality which implies that it does not, like solidarity, stem from personal feelings of empathy or commitment towards helping others:

Someone can favour equality without feeling a strong urge to become personally involved in the redistribution of wealth and power […] a belief in equality does not have to flow from emotions such as empathy [58].

Instead, egalitarian values are more *ideological* in nature, as they express a preferred vision of how to organise society. Egalitarianism also implies a much more far-reaching redistribution of income in a society in that it projects the goal of making all individuals equal in some sense. Solidarity signals a concern for and willingness to help others, but gives no indication of how much help is warranted or what the level of redistribution should be.

The rest of the paper is devoted to an empirical study of tax compliance in healthcare among individuals having VHI. In the next section the case of Sweden is presented, followed by a description of the methodological approach and the findings. The four types of motives for tax compliance identified (self-interest, group-interest, solidarity, and egalitarianism) are used to interpret the findings.

### Case study context – Healthcare in Sweden

Sweden has a tax-based healthcare system of NHS type. It is universal, as it offers all permanent residents access to a broad range of healthcare services on the same conditions, regardless of employment or ability to pay [59]. The system is decentralised in that it places responsibility for both funding and providing all healthcare services on 21 regional governments. The regions are free to set their own tax rates, which vary between 10.7 per cent and 12 per cent of a person’s income. User fees within the public system consist of co-payments for visits and hospital stays and are capped at the level of about €120 per year [60].

Healthcare provision in Sweden is predominantly public, as most hospitals and outpatient specialist care units are owned and managed by the regions. The regions can also purchase services from contracted private care providers. In 2018, about 20 per cent of all publicly funded visits to outpatient specialist care were conducted at privately operated clinics. Within the primary care sector, the share of publicly funded but privately provided care is higher, as about 40 per cent of all patient visits in 2020 were made with privately employed GPs [59]. Healthcare clinics operated by the regions themselves are not allowed to serve patients with VHI or who are paying out-of-pocket. Privately operated clinics, on the other hand, can receive both publicly and privately funded patients. Patients using VHI are directed to the private care market. The privately funded healthcare sector (i.e., healthcare services paid for by VHI or out-of-pocket) in Sweden is still very small, as it only accounted for about 1 per cent of total healthcare spending in 2019 [18]. In international comparisons, the Swedish healthcare system is often top-ranked on indicators of medical quality, but tends to get lower scores on patient information and patient involvement. Another challenge for the system is the long waiting time for certain treatments [59].

#### Voluntary health insurance in Sweden

A fairly recent development in the Swedish healthcare system is the growth of VHI. In 2001, about 100,000 individuals held insurance; in late 2025, the number had risen to 830,000, corresponding to almost eight per cent of the population [16, 61]. Swedish VHI is predominantly duplicating, as it covers services that are also provided by the public healthcare system. About 60 per cent of all VHI holders receive their insurance as a fringe benefit from their employer, while 30 per cent purchase it as a group-based insurance, often through unions, and 10 per cent buy it individually [61]. Individuals receiving VHI as an employment benefit usually pay a deductible when the insurance is used. The cost of VHI varies significantly depending on how it is obtained. For individuals purchasing VHI privately, the annual cost ranges between €200 and €600. For employer-funded VHI, most of the premiums are covered by the employer. However, since VHI is a taxable benefit, employees are required to pay income tax on approximately 60 per cent of the premium’s value. This means that, depending on the total premium, the effective cost for employees typically ranges from 26 to 43 € per month, which amounts to only 7–11 per cent of the regional healthcare tax, set at 10–12 per cent of income [62]. The insurance plans of Swedish VHI usually cover most forms of out-patient specialist care, less severe cancer treatments, rehabilitation services and therapy sessions with a psychologist. Emergency and intensive care are not covered, nor is reproductive health and highly specialised care. As a rule, pre-existing medical conditions are not covered [63].

Having VHI is more common among men in Sweden, but the gender differences are largely explained by occupation, as having VHI is more prevalent in male-dominated sectors. Almost one in three individuals employed within the finance, law, economic, and management consulting sectors receive VHI from their employer. In IT, media, and PR, the corresponding share is approximately 18 per cent. Most VHI holders are located in the metropolitan areas of Stockholm and Gothenburg. Furthermore, employer-funded VHI holders tends to have higher levels of education and income, are healthier, and do receive welfare benefits to a lesser extent compared to the average population [17]. VHI holders have also been found to sympathise more with political parties oriented towards the right on the political spectrum [19]. However, the causality remains unclear—whether VHI ownership influences political preferences or if these preferences are shaped by the employment sectors where VHI is common. Given that employer-funded VHI is concentrated in high-income, male-dominated sectors and metropolitan areas, VHI holders are likely to belong to groups less inclined to advocate for high taxes to support public healthcare.

## Methods

A qualitative approach with semi-structured interviews with VHI holders was chosen to investigate how the growth of VHI in Sweden affects the willingness to pay tax towards the public healthcare system and gain a deeper understanding of motives for tax compliance [64, 65]. This design was deemed most suitable as it allows for an in-depth exploration of complex attitudes and behaviours. Additionally, the lack of public registers or data on VHI holders in Sweden makes qualitative methods the most feasible and effective option for addressing the research questions. A purposeful, homogeneous sampling of respondents was used, with the aim of finding “typical” VHI holders [65]. In the Swedish case, this means white-collar workers with employer-funded VHI, working in areas of finance, consulting, or tech. These groups were selected as they represent the majority of VHI holders in Sweden and play a crucial role in maintaining the legitimacy of the public healthcare system, given their socioeconomic profile and significant contributions to public funding. Because both the majority of VHI holders and private healthcare clinics are found in the capital, Stockholm, respondents were recruited from this area. To be included in the study, respondents should have had the experience of using employer-funded VHI as well as having visited publicly funded healthcare services within the last couple of years. As there is no public register of VHI holders or companies offering VHI to their employees in Sweden, personal contacts and snowball sampling methods were used to recruit respondents.

In total, 20 interviews were conducted between February 2022 and October 2022. However, during the analysis, one interview was removed since it was found that the respondent did not fulfil all the criteria for inclusion. All interviews were conducted by the first author and carried out digitally with the video-meeting software Zoom and lasted between 26 and 83 min. Audio files were saved and transcribed verbatim. The interviews were conducted in Swedish (for translated interview-guide see Supplementary Material 1). Selected quotations have been translated into English by the authors. All but one of the respondents had a university degree and their ages ranged from 33 to 59, with a mean age of 45 years. Twelve were men and seven were women. The respondents were asked questions about their attitudes towards the public healthcare system and whether their willingness to pay tax towards it had changed as a result of having VHI. In cases of sustained willingness, respondents were asked about their motives for wanting to pay tax towards public healthcare.

**Fig. 1:**
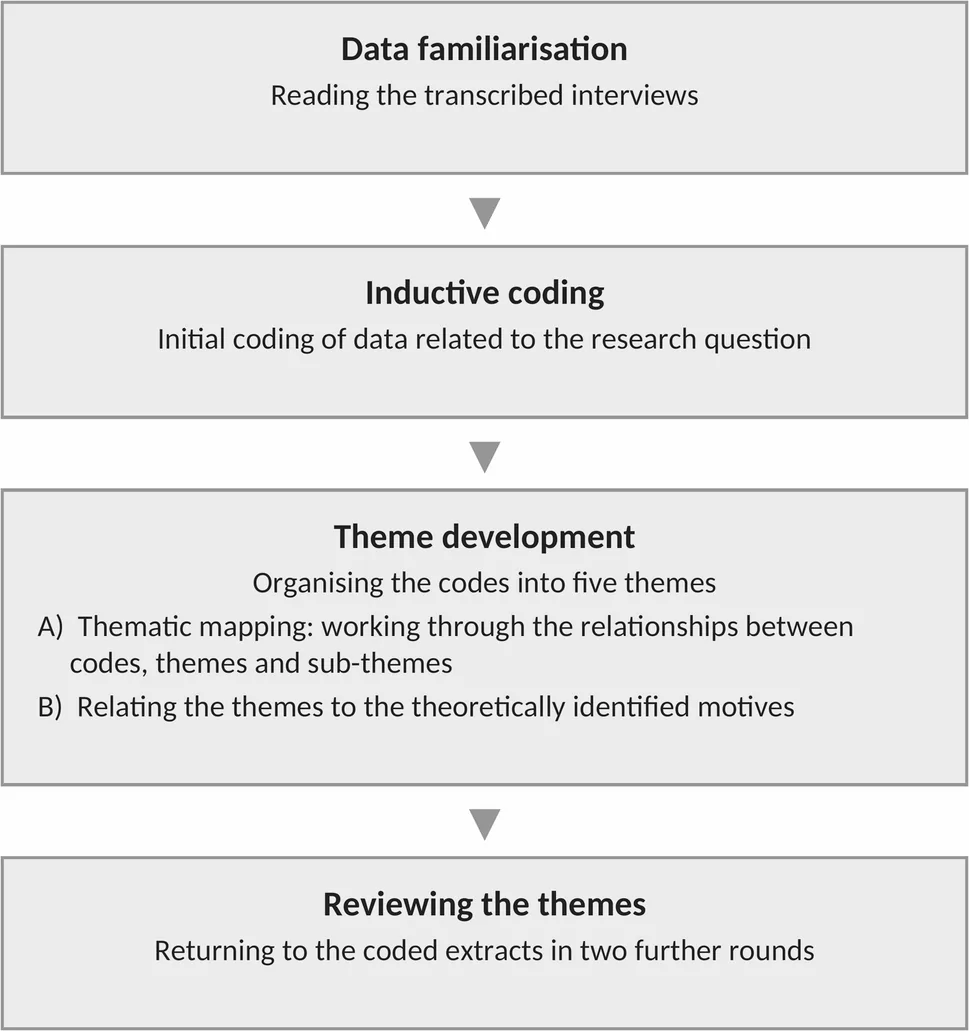
Overview of the analytical process, from familiarisation with the transcribed interviews and initial inductive coding, through the organisation of codes into themes and the visual mapping of their relationships, to the examination of the themes in relation to the theoretically identified motives and two further rounds of review

**Fig. 2:**
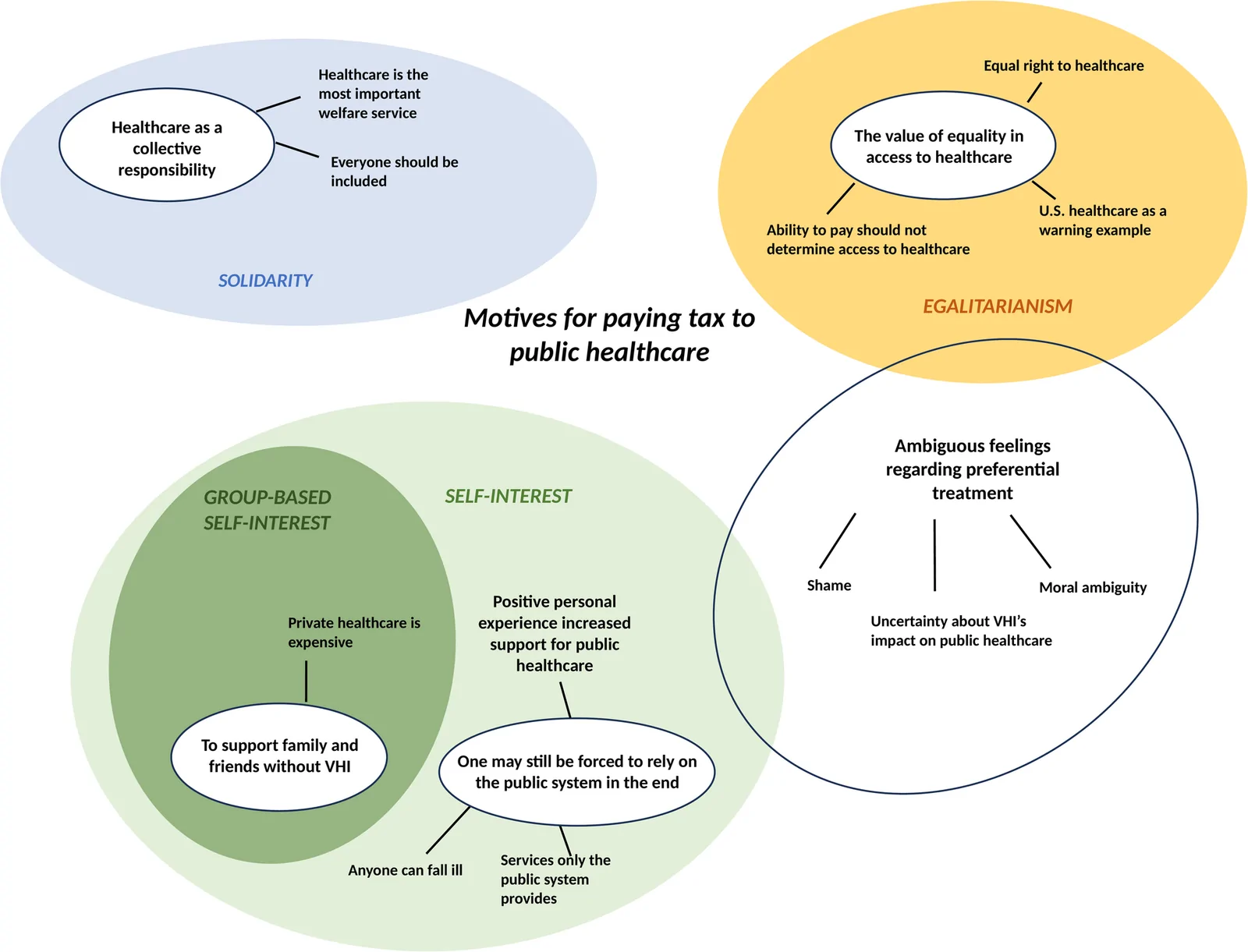
Thematic map of the five themes and their relation to the theoretical motives. The map shows how the five themes relate to the theoretically identified motives of solidarity, egalitarianism and self-interest, with group-based self-interest presented as a subcategory of self-interest. The fifth theme, ambiguous feelings regarding preferential treatment, is not a motive but the tension arising between the egalitarian and the self-interested motives, and is therefore placed across both theoretical motives

In order to analyse the material and answer the question regarding motives for tax willingness, thematic analysis was used [66, 67]. Inspired by Braun and Clarke, the analysis was conducted in several steps (see Fig. 1). The first author took primary responsibility for the initial steps of the analysis. First, the transcribed interviews were read for familiarisation. Second, an initial inductive coding was done. In this initial coding phase, only data related to the research question were coded [67]. In the third step, the codes were organised and developed into five themes. The entire author group regularly discussed the coding and the developing themes during project meetings, in order to consider alternative readings of the material rather than to verify the coding. The relationships between the codes, themes and sub-themes were then worked through by mapping them visually (Fig. 2, presented in the Results section). Constructing the map was an analytical step in its own right rather than simply a means of illustrating conclusions already reached. Mapping the themes in relation to one another required us to determine how they were connected, and in doing so the differences in their analytical level became apparent, as some reflected motives for paying tax while others reflected the conditions under which those motives were expressed.

The five themes were then examined in relation to the theoretically identified motives: solidarity, egalitarian values and self-interest, with group-based self-interest treated as a subcategory of self-interest rather than as a separate motive. This step allowed us to assess the relevance of the theoretical motives in the context of the material. In the fourth step, the themes were reviewed in two additional rounds to ensure their validity.

NVivo 1.5 software facilitated the thematic analysis, by organising and storing the coded transcripts. Ethical approval for the study (Dnr: 2020 − 01982) was granted by the Swedish Ethical Review Authority. All participants provided written consent to participate in the study.

## Results

After some of the introductory questions regarding their experiences of using their VHI, respondents were asked whether having the insurance had affected their willingness to pay tax towards the public healthcare system. Support for the public healthcare system was expressed throughout the interviews, and respondents did not describe their willingness to pay tax as being affected by their experience of holding VHI:

No, no, truly not. I would not want that. No feelings of: well, I already pay for this insurance, so I don’t want to pay double in tax. (Interview 20)

Another respondent explained that she saw VHI as a benefit that came with the employment, and not as a reason to pay less tax:

I just see this as a little bonus […] I don’t go around and think like, maybe I should pay less in tax. I don’t think like that. My brain doesn’t work like that. (Interview 10)

Resignation towards tax-paying in general could accompany this position, without the willingness to pay for healthcare being reduced:

I can’t affect my taxation. I can move to another municipality with lower levels of taxation. That’s it. So, I suck it up and pay double. (Interview 3)

Alongside the wish to continue paying tax, a preference for some form of tax compensation was also voiced:

No, [my willingness to pay tax is not reduced] […] but I think it is weird that you don’t get a tax-reduction, automatically, since I actually don’t [use and] burden the [public] healthcare […] but I can’t understand it. Two times [I pay taxes], since I also have to pay tax on this [employment-] benefit. That is sick. (Interview 15)

In sum, the willingness to pay tax towards the publicly funded healthcare system was described as sustained, and the insurance was not presented as having reduced it. Where reservations were voiced, they concerned taxation in general rather than the funding of healthcare.

### Motives for paying tax to public healthcare

#### Healthcare as a collective responsibility

One motive for continuing to pay tax towards publicly funded healthcare was the view that the provision of healthcare is an obligation borne by society as a whole rather than by individuals on their own behalf. Two aspects of that obligation recurred in the interviews: that *healthcare is the most important welfare service*, and that *everyone should be included* in it.

The first of these was evident in the way healthcare was set apart from public spending in general and placed ahead of other public commitments. Where respondents were critical of taxation as such and a large public sector, healthcare was still seen as a crucial public service that they wanted to support with their taxes:

Well, taxes in general one can have opinions about, but not regarding healthcare as such. If there is anything I am willing to pay for, it is healthcare and a few other things. I might have opinions about tax rates and such, but I think that is totally something else. (Interview 9)

It was spoken of as a unique service, one that a modern welfare society is expected to provide through the state, and as belonging among the government’s principal tasks. This held also for respondents who favoured a privately funded healthcare market as a complement to the public system; the complement was not presented as relieving the state of the responsibility.

A second aspect, that *everyone should be included*, concerned the scope of that responsibility. Here, the value of the public system lay in its guarantee that the whole population has access to healthcare, set against an alternative in which coverage would follow what each person could afford — an alternative under which the respondents themselves would been among benefited:

I think it is really important, because the insurance costs money and not everyone can have insurance. That is impossible. I am in the category of, well, not a very high earner. But we have good salaries and so on. So I want the whole of society to come along. If I were an egoist I would just want tax cuts and privatisation of everything … …And you want everyone to be included. So public healthcare, it is needed. Very much so. (Interview 3)

The support expressed here rests not on what the public system does for the respondent but on what it does for others.

Taken together, the two aspects express a view of healthcare as something society owes its members, and can be read as expressions of solidarity: a concern for the wellbeing of others and a willingness to contribute to it. The obligation described here is owed to the population at large, including people the respondents will never meet, which distinguishes it from the concern for family and friends discussed below. It is also distinct from the theme that follows, where what is at issue is not whether society bears an obligation but the principle according to which care should be allocated once it does.

#### The value of equality in access to healthcare

Another motive for tax compliance was that publicly funded healthcare is important to ensure an *equal right to healthcare* on the part of all individuals. A recurring specification of that right was that *the ability to pay should not determine access to healthcare*:

I think it is so important that everyone should have equal access [to healthcare]. I don’t think that you should go around with injuries that you don’t get help with because you don’t have money when you get sick. (Interview 5)

Related to the value of equal rights, respondents also expressed ideas about social justice, in which publicly funded healthcare was seen as essential for a fair distribution of healthcare. It was described as unfair, even “heart-breaking”, that people have unequal access to healthcare:

And I think it’s, like, totally heart-breaking to think that people get unequal healthcare. Or, like, that I could come to survive something that someone else could die from just because I happened to have a more well-paid job. (Interview 18)

Respondents also expressed a desire that publicly funded healthcare should receive even more funding:

I wish that they [publicly funded healthcare] would get more resources because I see how it is bleeding in a way. I wish we had a society where everyone gets equal healthcare. (Interview 12)

The *US healthcare system* was raised spontaneously throughout the interviews as a *warning example* of unequal access to healthcare that illustrates the importance of a public healthcare system. The emphasis on equal access to healthcare displayed an egalitarian view of how healthcare should be organised and came across in the interviews as an important motive for paying tax towards public healthcare.

#### One may still be forced to rely on the public system in the end

A third motive concerned the respondents’ own relationship to publicly funded healthcare: both the possibility that they might come to depend on it, and their experience of having already done so. Three aspects recurred: that *anyone can fall ill*; that there are *services only the public system provides*; and that *positive personal experience increased support for public healthcare*.

Respondents recognised that, although they were currently privileged and had good salaries, *anyone can fall ill* and be in need of public healthcare services:

Everyone can end up… Although you are sitting on a green branch now, who knows, things happen in life. Like no longer being able to pay. (Interview 8)

A second aspect concerned *services only the public system provides.* Respondents also noted that the VHI only covers a limited range of services. Emergency care, for instance, is only provided by the public healthcare sector:

[…] and on the day I have to go to the emergency room, then I have to go to the emergency room. So, it [publicly funded healthcare] has to work. Then you cannot call your voluntary health insurance. (Interview 10).

In a similar vein, respondents pointed out that highly specialised care is only offered at the public university hospitals and that this therefore was a motive for them to continue paying tax towards the public healthcare system:

And I believe that places like Karolinska [university hospital] are needed, truly. To produce another part of it, the specialised healthcare, where I’m also enrolled. Where all (doctors) I see have a PhD. (Interview 16)

A third aspect, that *positive personal experience increased support for public healthcare*, concerned respondents who had themselves made use of publicly funded services and described this as having strengthened their willingness to pay for them. The experience of child birth, a service not covered by VHI in Sweden, was mentioned in this way:

So, the children I have given birth to, for instance. I would pay any amount of tax to make that work. I’m almost starting to cry now. Cause it is so amazing how some [the midwives] work. So yes, I find that super important. (Interview 10).

Another respondent elaborated on how his extensive use of publicly funded healthcare due to a Type 1 diabetes diagnosis had affected his positive attitude and willingness to pay tax towards the public healthcare system.

These motives are clearly based on self-interest. Compared with the other themes in the material, however, they were expressed more briefly and with less elaboration.

#### To support family and friends without VHI

A further motive for paying tax towards public healthcare was concern for relatives and friends who did not have VHI:

Now when I get this [VHI] from my work […] I do not feel that I don’t want to pay more tax. Because I have a daughter, a mother and father who need [publicly funded healthcare]. They don’t have this [VHI]. They live on pensions. So, I think I can just continue to pay [taxes] and see this as a benefit. (Interview 17)

Concern for family members can be considered an expression of group-oriented self-interest — self-interest extended to those close to the respondent, rather than a motive of a different kind (see Fig. 2).

#### Ambiguous feelings regarding preferential treatment

Unlike the themes described above, this one does not offer a reason for paying tax. Rather, it highlights a tension between support for egalitarian principles and the use of private healthcare. Three aspects recurred: *moral ambiguity*, *shame*, and *uncertainty about VHI’s impact on public healthcare*.

The VHI was described as of great benefit to those who held it, and it was this benefit that generated the difficulty: the personal gain appeared to stand in conflict with the respondents’ view on how healthcare services ought to be distributed, namely on an egalitarian basis:

I think it is a bad system, with voluntary health insurance. But when you get this offer, for the individual, it is very beneficial. Like, if I need help, I know I can get it very quickly and the healthcare provider will shower me in follow-up visits (Interview 18).

The same tension appeared where respondents stated that they gladly paid tax for public healthcare while at the same time favouring tax reductions for VHI holders. One respondent expressed it as a feeling of shame:

Like I said, I almost feel ashamed that I had to use my voluntary health insurance. Like I wish it wasn’t needed, because I don’t think it’s right this way. But then I put myself first (laughter). Like all humans: put myself first. (Interview 5).

Uncertainty was also expressed about whether it is true that VHI tends to “crowd out” publicly funded patients and allow VHI-funded patients to skip lines. VHI was described as a good thing on the condition that it does not have this effect.

The ambivalence expressed here is not a motive in its own right but reflects a tension between the motives described above. Egalitarian values and self-interest were generally aligned when respondents discussed the public healthcare system in general. However, a tension emerged when employer-funded VHI was considered, as the benefits of VHI are linked to preferential access, whereas many respondents also emphasised equal access to healthcare as an important principle. Figure 2 therefore depicts this theme as overlapping the egalitarian and self-interested motives.

In summary, all of the theoretically identified motives for paying tax towards public healthcare were expressed in the interviews, though they were neither of the same kind nor equally prominent. Solidarity was articulated as a collective responsibility for the health of the population; egalitarianism as a principle governing how care should be allocated; and self-interest as the recognition that the respondents might themselves come to depend on the public system — a motive that also appeared in a group-based form, extended to family and friends without insurance. Egalitarian values, particularly the principle that everyone should have equal access to healthcare, were invoked most readily and elaborated at the greatest length, while motives based on self-interest were stated more briefly. The final theme was of a different kind. Rather than constituting a motive in itself, the ambivalence, and in one case shame, that respondents expressed at the thought of gaining faster access to care than others illustrates the tension between the egalitarian and the self-interested motives they held at the same time.

## Discussion

This study contributes to the literature on the consequences of privatisation on support for public healthcare, where little attention so far has been paid to the attitudes of individuals who have VHI. Using a qualitative approach, it offers a more detailed understanding of how employer-funded VHI holders account for their continued willingness to pay tax towards the public healthcare system.

One possible interpretation is that the high level of expressed support reflects an institutional feedback effect specific to the Swedish context. Institutional feedback theory holds that the design of welfare institutions shapes citizens’ attitudes towards welfare policies [68–72]; applied to Nordic countries, the argument is that universal systems, by including the whole population, generate higher support for redistributive policies than more targeted ones [20, 23]. The same logic may apply to healthcare, where equal access regardless of economic or social position is a prominent organising principle [21, 73]. Respondents’ accounts closely mirrored the discourse and normative values commonly voiced in relation to the Swedish healthcare system, which is consistent with a normative feedback mechanism [74] centred on a moral logic: universal policies are valued because they are perceived as just, providing equal treatment and fairness [20, 70].

Several limitations should be noted. First, the study rests on a limited number of interviews and is not intended to support statistical generalisation; its contribution is analytical rather than representative. However, the correspondence with patterns in an earlier national survey [15] increases confidence in the plausibility of the accounts. Second, support for tax-funded healthcare is a strongly sanctioned position in Sweden, so social desirability bias may have shaped what respondents were willing to express; stated willingness may also diverge from behaviour. Third, only employer-funded VHI holders were included. Because they bear no direct cost for their insurance, they face no personal financial trade-off between private and public provision. Those who purchase VHI privately may reason differently, and including them is an avenue for further research. Fourth, without a comparison group of individuals without VHI, the study cannot establish whether VHI holders differ from the general population, and its cross-sectional design cannot capture whether support weakens as coverage expands. Finally, the findings are tied to one institutional setting and should be transferred to differently organised systems only with caution.

Taken together, the findings indicate that holding employer-funded VHI did not, in these respondents’ accounts, undermine willingness to pay tax towards a universal, tax-funded healthcare systems. This does not support the assumptions in parts of the previous literature that VHI reduces support for and willingness to pay tax towards public healthcare [11, 40], although the present study cannot settle that question at the population level. Continued willingness was framed primarily in moral terms, with equal access for all citizens treated as a central value. One reading is that the universalistic and egalitarian construction of the Swedish healthcare system, where access to healthcare services is seen as a matter of social rights, helps sustain a sense of moral duty to contribute even among those who use private healthcare services alongside the public system.

## Conclusion

This study asked how employer-funded VHI holders in Sweden account for their continued willingness to pay tax towards the public healthcare system. The respondents expressed such a willingness and did not present their insurance as having reduced it, though some voiced resignation towards taxation in general. The motives they gave drew on solidarity, egalitarian values and self-interest, with egalitarian values invoked most readily and elaborated at the greatest length: equal access to healthcare was throughout the interviews raised as a reason for continuing to contribute. Alongside these motives, respondents expressed ambivalence about the faster access their insurance affords — a tension between the egalitarian principle they gave as their main reason for contributing and the advantage the insurance gives them. Support for publicly funded healthcare thus persisted among these respondents even as they used private services alongside the public system, but it was not held without friction.

## Supplementary Information

Below is the link to the electronic supplementary material.

Supplementary Material 1

## Data Availability

The data generated and analysed during the current study are not publicly available due to the necessity to ensure participant confidentiality. De-identified data can be provided from the corresponding author on reasonable request. Data is shared with caution due to the sensitive content in the material.
