# Supplementary material for: Why are voluntary health insurance holders willing to pay tax towards public healthcare? A qualitative study from Sweden
Source: BMC Health Serv Res. 2026 Sep 29;26:1322. doi: 10.1186/s12913-026-15708-3 (PMC13625401; doi:10.1186/s12913-026-15708-3)
Supplement: Supplementary file 1 [file 12913_2026_15708_MOESM1_ESM.docx]

*This is a revised interview guide where questions published in another study have been excluded. The questions were originally written in Swedish.*

**Interview Guide (2022-05-18)**

**Introductory Questions**

Let’s begin by clarifying the type of private health insurance you have:

- Did you receive your insurance through your employer?
- How long have you had it?
- Have you had a similar insurance policy before?
- Do you know which insurance company provides your policy?
- Approximately how many times have you used your insurance?
- Do you have an idea of what types of healthcare services are covered by your insurance?
- Is there anything that is not included in the coverage?
- Do you contribute financially towards the insurance yourself?
- If applicable: Why do you think your employer has chosen to provide this insurance for you?
- Education / professional background
- Age

For the sake of clarity, what we have discussed so far refers to **private healthcare services accessed through private health insurance**. This is distinct from the **publicly funded healthcare system**, which is financed through taxes and managed by the regional authorities (formerly county councils). You are likely aware that private healthcare providers also operate within the publicly funded system—for instance, companies such as Praktikertjänst and Capio run GP surgeries that are accessible to all patients within the public system. However, in this interview, I will refer to **publicly funded healthcare** when discussing services financed through taxation, and this should be seen as separate from the healthcare you access via private insurance.

- Do you know whether your registered GP surgery is publicly operated by the regional authority or privately run?

**Taxation and Support for the Public Healthcare System**

I am interested in understanding how important you consider the **publicly funded healthcare system**, given that you also have access to private healthcare through your insurance.

1. How important is it to you that we have a publicly funded healthcare system?
   - **Follow-up:** Why do you think it is important?
2. Do you think your access to private health insurance has influenced your view of the importance of the public healthcare system?
   - If so, in what way? Can you recall how you thought about it before?

The public healthcare system is primarily financed through taxation.

3. Do you think your attitude towards paying taxes for public healthcare has changed since you gained access to private healthcare through your insurance?
   - Why? / Why not?
   - Why do you think it is (or is not) worthwhile to pay taxes for public healthcare?
   - Would you prefer healthcare to be financed in a different way, for instance through higher patient fees or increased reliance on private insurance?
4. More broadly, what is your opinion on the system of private health insurance?
   - Since private insurance can provide shorter waiting times for some individuals compared to those who rely solely on the public system, do you see this as a problem?

**Closing the Interview**

We are about to finish, but before we do, I would like to summarise what I have heard you say and give you the opportunity to clarify any points or add anything you feel has been missed.

Thank you very much for your time.
